# Do Social Connections Buffer Loneliness Associated With Living Alone?

**DOI:** 10.1093/geroni/igab046.1777

**Published:** 2021-12-17

**Authors:** Markus Schafer, Haosen Sun, Jin Lee

**Affiliations:** University of Toronto, Toronto, Ontario, Canada

## Abstract

The growth of solo living has important implications for the rising “loneliness epidemic” among older adults. This study considers whether two forms of social connectedness—extra-household core discussion networks and formal social participation—buffer the loneliness associated with living alone. Our study uses data from two surveys (National Social Life, Health, and Aging Project; Survey of Health, Ageing and Retirement in Europe) encompassing 20 developed Western countries in 2009/2010 and 2015/2016 (n = 110,817). Harmonizing measures across data sets, we estimate survey-specific and pooled linear regression models with interaction terms. Results indicated that high levels of social connectedness only moderately buffer the loneliness associated with living alone in later life. Findings were largely consistent across regions of Europe and the United States, though the buffering patterns were most robustly identified for widowed solo dwellers. Taken together, the results suggest that extra-household connections are partial compensators, but do not seem to fully replace the ready companionship afforded by residential co-presence in later life. Future research is needed to understand whether the efficacy of compensatory connections differs by gender, race/ethnicity, and across more diverse global regions.

